# Bibliographical Notices

**Published:** 1845-05

**Authors:** 


### BIBLIOGRAPHICAL NOTICES.

*Bulletin of Medical Science*, Edited by JOHN BELL, M.D., &c., Nos. 1, 2, 3, Vol. 3. Philadelphia, Barrington & Haswell.

This is a monthly journal of 36 pages, 8vo., and from an examination of the numbers before us, we have formed a high opinion of its merits, finding it among the most interesting and valuable of our exchanges.

*Twenty-fourth Annual Report of the Bloomingdale Asylum for the Insane, for the Year* 1844. From PLINY EARLE, M.D., Physician to the Institution.

The subject of "Mental Maladies," and their treatment, is now occupying the attention of a large portion of the profession, and notwithstanding the great progress which has been made in relation to the pathology of these affections, every year adds something to our knowledge respecting them. The Bloomingdale Asylum ranks with the oldest in the country, and in all respects is well conducted. It is a branch of the New York Hospital, and has shared largely in the munificence of the state government.

We have not time, nor would it be altogether consistent with the object of this journal, to enter into a consideration of the various topics of interest presented in this Report, but we would call the attention of the medical profession to the importance of commencing one or more retreats, for the reception of the insane, in this State. Our sister State, Indiana, although not more favorably situated in a financial point of view than Illinois, has already taken this subject in hand, urged forwards by the efforts of Dr. Jno. Evans, of Attica, who is now at the east examining different establishments, with reference to planning one for that State. Here, too, the first impulse must come from physicians, and we know of nothing in which a more valuable service could be rendered to community, and a well deserved reputation established, than by devotion to this object.

